# Splenic artery loops: Potential splenic plexus stimulation sites for neuroimmunomodulatory‐based anti‐inflammatory therapy?

**DOI:** 10.1002/ca.23643

**Published:** 2020-07-06

**Authors:** Cindy G. J. Cleypool, Dyonne Lotgerink Bruinenberg, Tom Roeling, Eric Irwin, Ronald L. A. W. Bleys

**Affiliations:** ^1^ Department of Anatomy, Division of Surgical Specialties, University Medical Center Utrecht Utrecht University The Netherlands; ^2^ Galvani Bioelectronics Stevenage UK; ^3^ Department of Surgery University of Minnesota School of Medicine Minneapolis Minnesota USA

**Keywords:** anti‐inflammatory pathway, neuro‐immunomodulation, spleen, sympathetic nervous system, splenic artery loops, splenic innervation, splenic plexus

## Abstract

**Introduction:**

The splenic plexus might represent a novel neuroimmunomodulatory therapeutic target as electrical stimulation of this tissue has been shown to have beneficial anti‐inflammatory effects. Tortuous splenic artery segments (splenic artery loops), including their surrounding nerve plexus, have been evaluated as potential stimulation sites in humans. At present, however, our understanding of these loops and their surrounding nerve plexus is incomplete. This study aims to characterize the dimensions of these loops and their surrounding nerve tissue.

**Materials and Methods:**

Six formaldehyde fixed human cadavers were dissected and qualitative and quantitative macro‐ and microscopic data on splenic artery loops and their surrounding nerve plexus were collected.

**Results:**

One or multiple loops were observed in 83% of the studied specimens. These loops, including their surrounding nerve plexus could be easily dissected free circumferentially thereby providing sufficient space for further surgical intervention. The splenic plexus surrounding the loops contained a significant amount of nerves that contained predominantly sympathetic fibers.

**Conclusion:**

The results of this study support that splenic artery loops could represent suitable electrical splenic plexus stimulation sites in humans. Dimensions with respect to loop height and width, provide sufficient space for introduction of surgical instruments and electrode implantation, and, the dissected neurovascular bundles contain a substantial amount of sympathetic nerve tissue. This knowledge may contribute to further development of surgical techniques and neuroelectrode interface design.

**Abbreviations**
BSA: bovine serum albumin
CAIP: cholinergic anti‐inflammatory pathway
CGRP: calcitonin gene related product
ChAT: choline acetyl transferase
IQR: inter quartile range
IR: immune reactive
LGEA: left gastroepiploic artery
LPR: liquid permanent red
PA: pancreatic arteries
PGP: protein gene product 9.5
RT: room temperature
SA: splenic artery
SGA: short gastric artery
SPS: splenic plexus stimulation
TB: terminal branches
TBS: tris buffered saline
TH: tyrosine hydroxylase
VNS: vagus nerve stimulation

## 1 INTRODUCTION

Neural regulation of the immune system, also known as neuro‐immunomodulation, has gained attention for its potential as a new anti‐inflammatory therapeutic method. Various neural pathways are suggested to be involved in neuro‐immunomodulation with one of these pathways, the cholinergic anti‐inflammatory pathway (CAIP), also known as the inflammatory reflex, having been studied most extensively (Chavan & Tracey, 2017). The CAIP comprises an efferent neural pathway that dampens the systemic inflammatory response via the spleen and involves sequential activation of the efferent vagus nerve and sympathetic splenic nerve tissue (Pavlov & Tracey, 2017; Reardon, 2016).

The anti‐inflammatory effect of the CAIP has been studied using electrical stimulation of the vagus nerve (VNS) in both rodent and human inflammatory models (Bassi et al., 2017; Borovikova et al., 2000; Ghia, Blennerhassett, Kumar‐Ondiveeran, Verdu, & Collins, 2006; Koopman et al., 2016). Although stimulation of the CAIP, using VNS, has been shown to modulate inflammation, VNS is associated with a variety of side effects including cough, hoarseness and paresthesia (Ben‐Menachem, 2001; Cristancho, Cristancho, Baltuch, Thase, & O'Reardon, 2011). Furthermore, controversy remains as to whether the anti‐inflammatory effect of VNS is the result of activation of efferent or afferent vagal nerve fibers, since vagal efferent neurons neither synapse with splenic sympathetic neurons nor drive their ongoing activity (Bratton et al., 2012). If the observed anti‐inflammatory effect of VNS is the result of afferent stimulation, activation of the CAIP most likely is preceded by an unknown central reflex and the distal part of the CAIP, represented by post‐ganglionic sympathetic fibers of the splenic plexus (Kees, Pongratz, Kees, Schölmerich, & Straub, 2003; Niijima, Hori, Aou, & Oomura, 1991; Vida, Peña, Deitch, & Ulloa, 2011), may serve as a more direct stimulation target.

Electrical stimulation of the splenic plexus, in rats, has been shown to reduce levels of systemic tumor necrosis factor (TNF) after administration of the endotoxin lipopolysaccharide (Kees et al., 2003; Vida et al., 2011) and mitigated clinical symptoms in a mouse model of rheumatoid arthritis (Guyot et al., 2019). Therefore, electrical splenic plexus stimulation (SPS) in humans might hold potential as an anti‐inflammatory therapy and knowledge on the anatomy of this specific nerve tissue is essential for further therapeutic design.

The human spleen is reported to be innervated by sympathetic nerve fibers (Heusermann & Stutte, 1977; Kuntz, 1953; Verlinden et al., 2018). These fibers originate in the celiac ganglia and run in a perivascular nerve plexus around the celiac trunk and subsequently the splenic artery (SA) as they travel towards the splenic hilum (Mitchel, 1956). At the hilum the SA ramifies and the surrounding splenic plexus continues around these smaller branches entering the splenic pulp (Mitchel, 1956). Because of this perivascular plexus‐like configuration, the optimal neuro‐electrode interface may require a circumferential device around the SA neurovascular bundle. Due to the close proximity of the SA to the pancreas and the fact that it can even be embedded within the pancreatic parenchyma, concern has been raised about the risk of damaging or irritating pancreatic tissue during circumferential dissection of the SA neurovascular bundle in preparation for electrode application. To address this concern, segments of the SA with increased tortuosity where the artery moves away from the pancreas (Michels, 1942; Sahni, Jit, Gupta, Gupta, & Harjeet, 2003) have been considered as intervention points. These segments, further referred to as SA loops, may represent safe intervention sites for electrical SPS.

In order to assess the feasibility of SA loops as electrical SPS sites in humans, morphological information on these structures is needed to inform surgical and engineering activities. Although the human SA and its tortuosity has been previously studied (Michels, 1942; Sahni et al., 2003), these studies do not supply all required information for neuroelectrode interface design and surgical implantation technique development. Therefore, this study aims to provide both descriptive and quantitative information on the presence, number, location, and dimensions of SA loops, and, on the presence, amount, type and organization of their perivascular nerve tissue.

## 2 MATERIALS AND METHODS

Six human cadavers were used for this study: four females and two males with a median age of 93 years at death (interquartile range [IQR]: 79–98). Whole body preservation was accomplished by arterial perfusion with 3% formaldehyde. The spleen, stomach, pancreas, splenorenal ligament, greater omentum, and gastrosplenic ligament were removed in toto and stored in 2% formaldehyde until further dissection. All bodies entered the Anatomy department through a body donation program and by prior written informed consent, the donors allowed their bodies to be used for educational and research purposes.

### 2.1 Macroscopic examination

**FIGURE 1:**
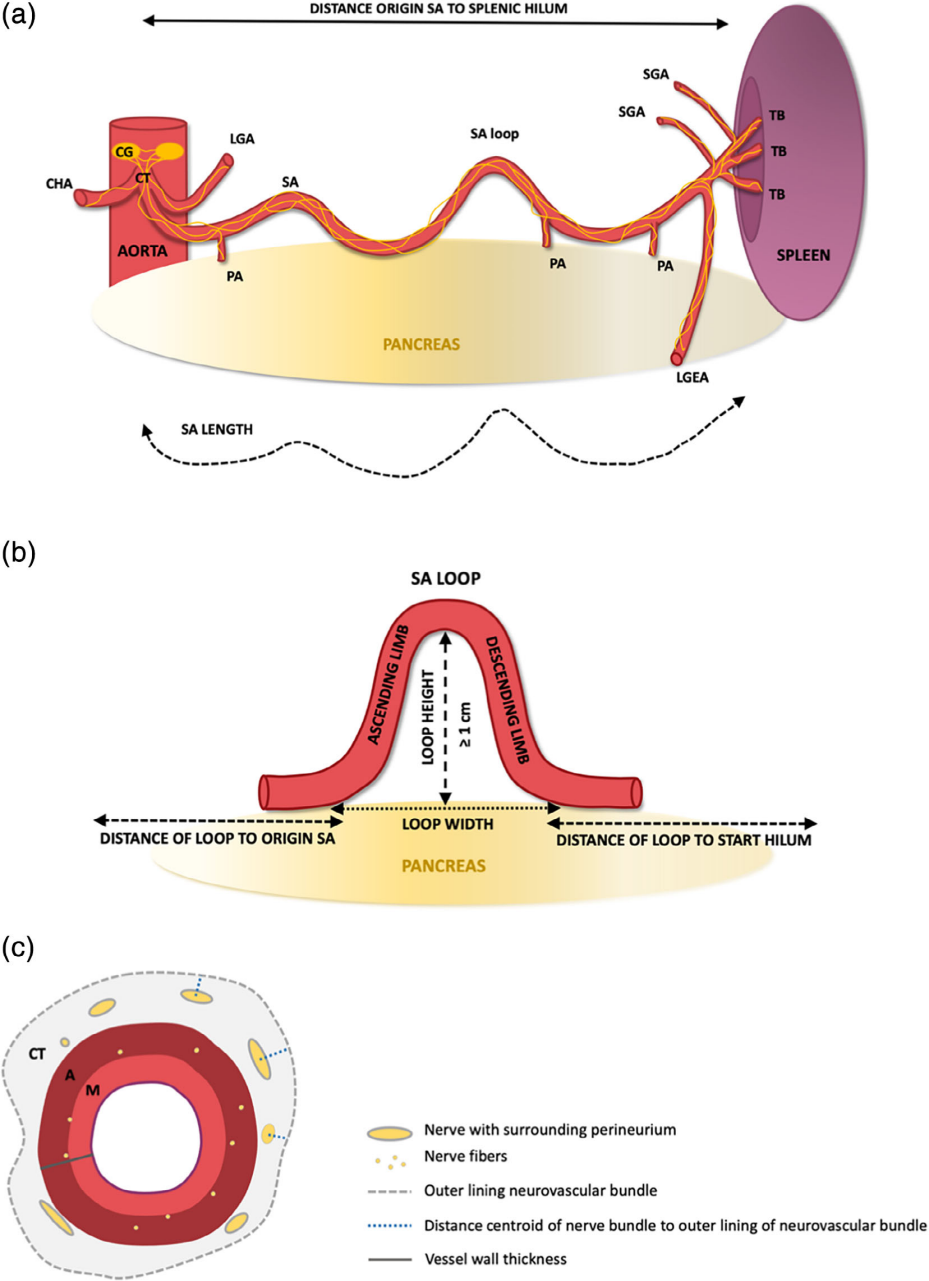
Schematic representation of investigated macro‐ and microscopic morphometric parameters. (a) Macroscopic parameters SA. Distance of the spleen to the origin of the SA, measured in a straight line from the start of the splenic hilum to the origin of the SA at the celiac trunk. SA length, was measured by placing a cord alongside the SA, from the origin of the SA at the celiac trunk to the start of the splenic hilum. CT, celiac trunk; LGA, left gastric artery; LGEA, left gastroepiploic artery; PA, pancreatic artery; SGA, short gastric artery; TB, terminal branch. (b) Macroscopic parameters SA loop. Loops are defined as parts of the SA that extend away from the pancreas with a height of minimal 1 cm, measured from the inside of the apex of the loop and the surface of the pancreas. The loops contain a proximal ascending and a distal descending limb. The width of the loop is measured between the start of the ascending—and descending loop. Loop location is determined as the distance of the ascending limb to the origin of the SA and the distance of the descending loop to the start of the hilum. (c) Microscopic parameters SA loop and its surrounding nerve tissue. Cross section of a SA loop neurovascular bundle, containing the SA with its three layers (I, M and A) and its surrounding connective tissue. Nerves are present in the connective tissue layer (CT) and smaller nerves and fibers can be found in the adventitia until the tunica media. CT, connective tissue; I, tunica intima; A, tunica adventitia; M, tunica media [Color figure can be viewed at wileyonlinelibrary.com]

Dissection was performed macroscopically. Occasionally a surgical microscope (Topcon, OMS75, Tokyo, Japan) was used for more detailed dissections. Descriptive data were collected on the SA, its loops and surrounding structures. Quantitative data were collected on the SA length, the distance of the spleen to the origin of the SA, the SA branching pattern and the number of SA loops. For each SA loop, the height and width of the loop, and the diameter of the ascending and descending limbs of the loop were determined (Figure 1a,b). Distances and diameters were measured using a ruler and caliper, respectively.

### 2.2 Microscopic examination

Following dissection and investigation of macroscopic parameters, part of SA loops and their surrounding nerve plexus, from now on referred to as the SA loop neurovascular bundle, were removed from halfway up the ascending limbs of the loops and processed for microscopic examination.

### 2.3 Preparation of tissue sections

All samples were decalcified with EDTA for 6 days (12.5% EDTA in distilled water, pH 7.5) and sequentially placed in increasing percentages of ethanol, xylene and paraffin. Samples were embedded in paraffin and cut on a microtome (Leica 2050 Super Cut, Nussloch, Germany) and 5 μm thick cross sections of one level of the neurovascular bundles were collected on glass slides, airdried and subsequently heat fixed for 2 hr on a slide drying table of 60°C (Medax, 14801, Kiel, Germany). Adjacent sections of each sample were stained with antibodies raised against protein gene product 9.5 (PGP9.5), tyrosine hydroxylase (TH), choline acetyltransferase (ChAT) and calcitonin gene‐related peptide (CGRP), staining for general, sympathetic, parasympathetic, and afferent nerve tissue, respectively.

### 2.4 Immunohistochemistiry

Sections were deparaffinated and rehydrated followed by 20 min of antigen retrieval in 95°C citrate buffer on a hot plate. After washing in Tris‐buffered saline (TBS) with 0.05% tween, sections were pre‐incubated with 5% normal human serum in TBS for 10 min, followed by incubation with primary antibodies (rabbit anti‐PGP; DAKO Z5116, Glostrup, Denmark) 1:2,000 for 48 hr at 4°C, rabbit anti‐TH (Pel Freez P40101, Rogers, USA) 1:1,500 overnight at room temperature (RT), goat anti‐ChAT (Milipore AB144P, Burlington, USA) 1:50 overnight at 4°C, or mouse anti‐CGRP (Sigma C7133, Saint Louis, USA) 1:1,500 overnight at 4°C) in TBS with 3% bovine serum albumin. Sections were washed with TBS‐0.05% Tween several times and incubated for 30 min at RT with Brightvision Poly‐AP Goat‐anti‐Rabbit (ImmunoLogic, Duiven, the Netherlands) (PGP and TH) or Brightvision Poly‐AP Goat‐anti‐Mouse (ImmunoLogic, Duiven, the Netherlands) (CGRP). In case of staining with ChAT, sections were incubated for 60 min with biotinylated horse anti goat (Vector, Burlingame, USA) 1:500 at RT followed by washing with TBS‐0.05% Tween and incubation with Streptavidin‐AP (Vector, Burlingame, USA) 1:500 for 60 min. After incubation with secondary antibodies, all sections were washed with TBS and incubated with liquid permanent red (LPR) (DAKO, Glostrup, Denmark) for 10 min. Tissue sections were then washed with distilled water and counterstained with hematoxylin, airdried at 60°C for 90 min and cover slipped using Entellan (Merck, Darmstadt, Germany). Negative controls were obtained by incubation with TBS‐3%BSA without primary antibodies. Human vagus nerve sections were included as a positive control for general, afferent, and parasympathetic nerve tissue and sympathetic trunk slides for sympathetic nerve tissue.

### 2.5 Microscopic imaging

Brightfield and fluorescent single images and stitched overview images (also known as tile scans) were captured of the same sample using a DM6 microscope with a motorized scanning stage, a DFC7000 T camera and LASX software (all from Leica, Nussloch, Germany). Single images were obtained with various magnifications whereas the ×20 objective was used for tile scans.

### 2.6 Image analysis

Tile scans of cross sections of SA loops stained with different markers were analyzed in Fiji (ImageJ with additional plugins) (Schindelin et al., 2012). The outer and inner lining of the total neurovascular bundle (SA with its surrounding connective tissue and nerve plexus) and, all perivascular nerves with their surrounding perineurium, were selected manually. The perimeter (circumference) of the total neurovascular bundle and its tissue area, the number of nerves, nerve diameter (short axis), nerve area (including perineurium), vessel wall thickness, diameter of the total neurovascular bundle and distance of the nerves centroid (geometric center) to the outer lining of the neurovascular bundle were measured (Figure 1c).

A threshold was set allowing to select all TH‐immune reactive (IR) tissue inside the nerves of the splenic plexus and to calculate their (pixel) area. The area of sympathetic nerve tissue was then calculated for each loop and the amount of sympathetic nerve tissue was then expressed as area% with respect to the total tissue area of the loops.

### 2.7 Statistics

Descriptive statistics were used to display quantified data which were calculated in SPSS.

## 3 RESULTS

### 3.1 Macroscopic morphometric data

#### 3.1.1 *Number, orientation, and relation of SA loops with surrounding structures*

**FIGURE 2:**
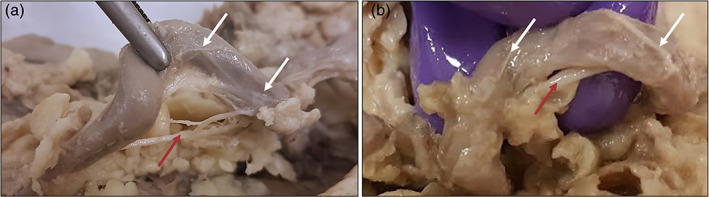
Splenic plexus in relation with SA loops. Nerves visible on the SA mostly follow the SA at the loop, but occasionally nerves were observed to run below the inner curvature of the loop and continue with the SA after the loop. (a) SA loop with nerves following the loop, but also running below the loop. (b) SA loop showing one relatively thick nerve (arrow) running just below the inner curvature of the loop and smaller nerves running with the loop [Color figure can be viewed at wileyonlinelibrary.com]

A total of eight loops were found in six specimens. A median of 1 (IQR: 1–2) loop was found per cadaver. One individual did not have any loops, three individuals contained one loop, one individual contained two loops and one individual contained three loops. Seven out of the eight observed SA loops extended in a dorso‐cranial direction with respect to the pancreas whereas only one loop extended in a more ventro‐caudal direction. All loops were covered with adipose tissue, a layer of loose connective tissue, and an irregular network of firm white strands (Figure 2), which, on histological analysis, were shown to represent the splenic plexus. In all cases parts of the loops could be visualized prior to dissection. However, in specimens with less adipose tissue loops were more easily identified. Loops were easily separated from surrounding adipose‐ and connective tissue, using blunt dissection, whereas the splenic plexus remained attached.

**FIGURE 3:**
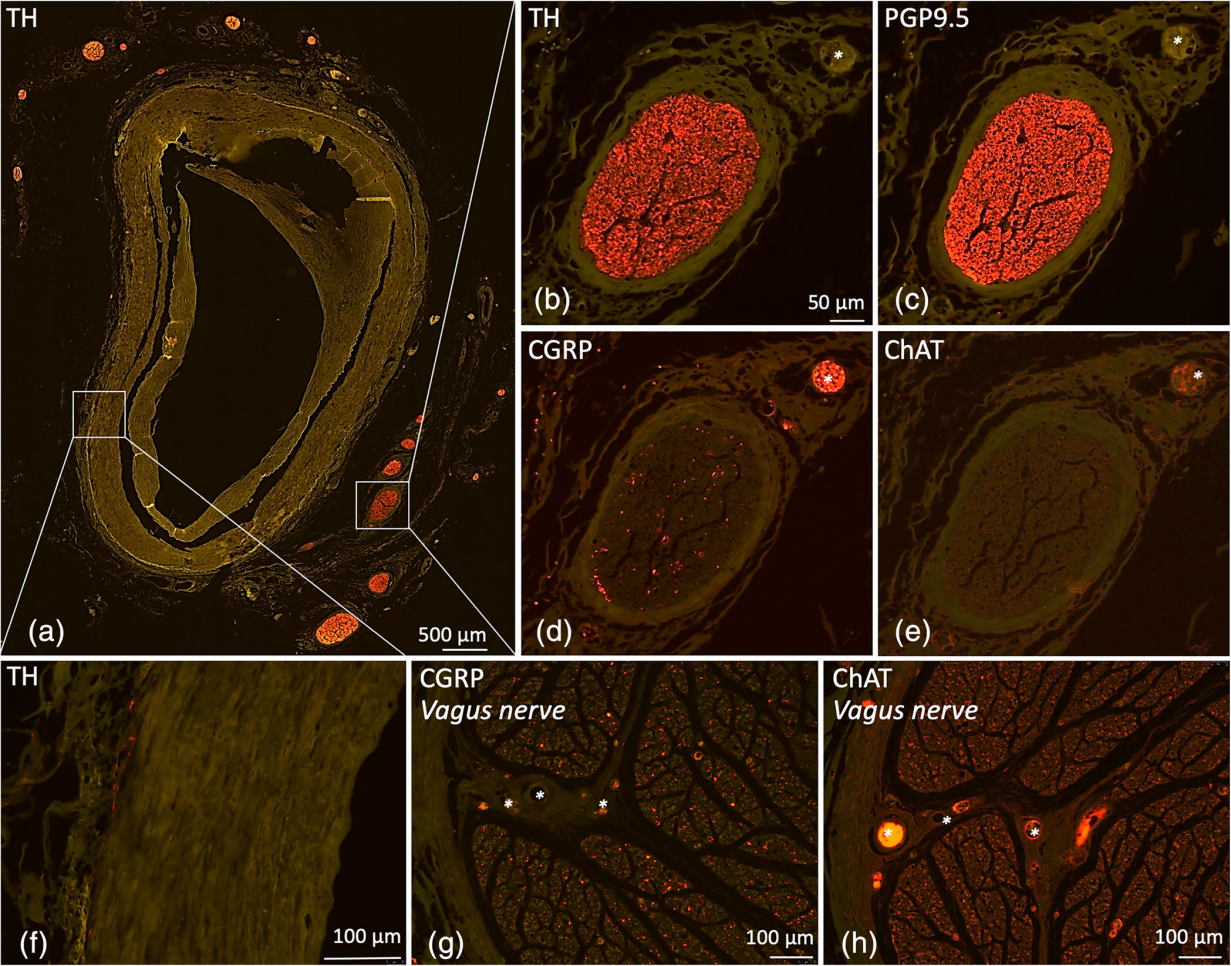
Microscopic images of SA loop related splenic plexus stained with various nerve markers. (a) Overview image of a SA loop cross section stained with TH, showing the organisation of sympathetic nerves of the splenic plexus. (b–e) Higher magnifications of an individual nerve stained for TH, PGP, CGRP, and ChAT. PGP9.5 and TH‐IR patterns are comparable, suggesting the splenic plexus to predominantly be composed of sympathetic nerve fibers. Only a few fibers are stained with CGRP and even less with ChAT, suggesting little sensory and parasympathetic nerve fibers, respectively. CGRP and ChAT‐IR was also observed inside blood vessels (asterisk in d, e, g, and h) and ChAT‐IR was observed in blood vessel walls. (f‐h) Positive controls [Color figure can be viewed at wileyonlinelibrary.com]

In the majority of the studied specimens, the splenic plexus as a whole remained in close proximity to the adventitial surface of the SA while running along the course of the SA and its loops (Figure 3a). In only one case a discrete nerve was observed to cross from one limb to the other, at the lowest part of the loop, and was found to rejoin and continue with the SA after the loop (Figure 3b). The thickness and patterning of the nerves of the splenic plexus varied within each specimen and between specimens. There was no obvious predominant topographical orientation of nerves with respect to the dorsal, ventral, cranial or caudal sides of the vessel wall.

#### 3.1.2 *SA length, tortuosity, and loop location*

The median length of the SA, measured from the origin of the SA to the spleen by placing a cord along the surface of the SA, was 18.9 cm (IQR: 12.9–21.1 cm) while the linear distance between the origin of the SA and the spleen was 11.3 cm (IQR: 9.6–13.5 cm), suggesting a median tortuosity of 54% (IQR: 44.3–62.8%). All loops were located at the pancreatic part of the SA. The median distances from the start and ending of the loops to the origin of the SA and splenic hilum, was 6.1 cm (IQR: 3.7–8.4 cm) and 3.8 cm (2.1–6.4 cm), respectively. Table 1 contains an overview of the macroscopic morphometric data.

**TABLE 1:** Quantitative macroscopic morphometric data on the SA and its loops

Parameter	Median	First quartile	Third quartile
SA (*n* = 6)			
SA length (cm)	18.9	12.98	21.05
Distance of origin SA to spleen (cm)	11.25	9.63	13.5
SA elongation (%)	54	44.25	62.75
Number of branching PAs	5	4	5
Number of branching SGAs	3	2	5
Number of SA TBs	4	3	7
Number of loops	1	1	2
SA loops (*n* = 8)			
Loop width (cm)	2	1.5	2.45
Distance of loop to origin SA (cm)	6.05	3.65	8.38
Distance of loop to spleen (cm)	3.75	2.13	6.43
Loop height (cm)	1.15	1.00	1.65
Diameter SA limb (cm)	0.40	0.45	0.50

#### 3.1.3 *Height, width, and diameter of SA loops*

The average loop height, measured as the distance between the inner curve of the apex of the loop and pancreas, was 1.2 cm (IQR: 1.0–1.7 cm). Loop width, measured as the distance between the ascending and descending limbs of the SA loop at their origins, was 2.0 cm (IQR: 1.5–2.5 cm). The median diameter of loop limbs, irrespectively if these were the ascending or descending limbs, was 0.40 cm (IQR: 0.45–0.50 cm).

#### 3.1.4 *SA course and branching pattern*

In all six specimens the SA originated from the celiac trunk. Its course was mostly suprapancreatic but SA segments were also found to travel in retropancreatic, intrapancreatic, or anteropancreatic locations. A number of branches were shown to originate from each SA: One left gastroepiploic artery (LGEA) and a median of five pancreatic arteries (PAs; IQR: 4–5), three short gastric arteries (SGAs; IQR: 2–5) and four terminal SA branches (TBs; IQR: 3–7). Branching arteries were found to originate from each of the eight observed loops. One‐to‐two PAs were shown to arise from three of the loops. One‐to‐two SGAs were found to arise from five loops and the LGEA was found to arise from two loops.

### 3.2 Microscopic morphometric data

All neurovascular bundle cross sections of SA loops demonstrated a blood vessel with clearly discernible tunica adventitia, media and intima. In some specimens the tunica intima was thickened due to atherosclerotic calcifications. The adventitial layer and surrounding connective tissue were loosely organized and continuous with each other, and no clear borders between the two were observed. Small nerves were present in the tunica adventitia of all specimens, and were observed until the tunica media (Figure 3). In this continuous connective tissue compartment multiple nerves of variable sizes and shapes, were identified around the circumference of the artery. These nerves appeared randomly distributed and oriented around the artery. Thus, in cross‐sections of the SA the surrounding nerves, depending on their cutting plane, appear as round, ellipsoid or elongated structures. In ellipsoid and elongated structures, the perimeter and surface area are not representative measures for the amount of excitatory nerve tissue. In order to correct for these misassumptions, the short axis of these structures is allowed to be used to recalculate perimeter and surface area (Weibel, 1979).

The median perimeter of the total neurovascular bundle was 26.46 mm (IQR: 19.24–28.78 mm), its tissue area (lumen area excluded) 31.49 mm^2^ (IQR: 15.83–35.06 mm^2^) and its vessel wall thickness, measured at locations without calcifications, was 659 μm (IQR: 470.8–774.5 μm). The median number of nerves surrounding a SA loop was 28 (IQR: 21–34.5), with a median nerve diameter and tissue area (both including the perineurium) of 33 μm (IQR: 18–87 μm) and 788 μm^2^ (IQR: 203–5,324 μm^2^), respectively. The average distance of the centroid (geometric center) of a nerve to the outer connective tissue lining of the neurovascular bundle was 446 μm (IQR: 247–769 μm). Table 2 contains an overview of the microscopic morphometric data.

**TABLE 2:** Quantitative microscopic data on SA loops and surrounding nerve tissue

Parameter	Median	First quartile	Third quartile
SA loops			
Perimeter of neurovascular bundle (mm)	26.46	19.24	28.78
Tissue area of neurovascular bundle (mm^2^)	31.49	15.83	35.06
Thickness of vessel wall (μm)	659	470.80	774.50
Nerve tissue			
Number of nerves/loop	28	21	34.50
Diameter of nerves (short axis) (μm)	33	18	87
Distance of nerves to the outer lining neurovascular bundle (μm)	446	247	769
Tissue area of nerves (including perineurium) (μm^2^)	788	203	5,324
TH‐IR nerve tissue in nerves (μm^2^)	297	36	2024
TH‐IR area/loop (μm^2^)	88,190	10,792	197,391
TH‐IR tissue/loop (%)	0.34	0.06	0.60

During a qualitative assessment of all tissue slides it was noticed that the amount and location of PGP9.5 and TH‐IR tissue inside nerves appeared equivalent, suggesting the observed nerves contain predominantly sympathetic nerve tissue (Figure 3). This observation was quantitatively confirmed by digitally analyzing various microscopic images of both PGP9.5 and TH stained tissue slides, which resulted in comparable values. As such, further studying of PGP9.5 stained sections would not provide additional information and therefore no further quantitative analysis was performed on these slides. The median area of TH‐IR nerve tissue per nerve was 297 μm^2^ (IQR: 36–2,024 μm^2^) and the median total area of TH‐IR tissue surrounding a SA loop was 88,190 μm^2^ (IQR: 10,792–197,391 μm^2^), which was 0.34% (IQR: 0.06–0.60%) of the total neurovascular bundle tissue area.

CGRP and ChAT staining were performed on adjacent slides to study whether nerves contained sensory or parasympathetic nerve fibers, respectively. A small amount of CGRP‐IR was observed in nerves. CGRP‐IR was mostly observed inside small blood vessels whereas merely few CGRP‐ IR areas were observed in the nerve tissue itself (Figure 3c). ChAT‐IR tissue was observed in nerves as well, but mainly in the wall of small blood vessels and not in structures that could represent nerve fibers (Figure 3d). No further quantitative analysis was performed on CGRP and ChAT stained slides.

## 4 DISCUSSION

This study shows that one or multiple SA loops are present in the majority of studied subjects and the splenic plexus surrounding these loops contains a substantial amount of predominantly sympathetic nerve tissue.

### 4.1 Presence, number, and location of loops

In the present study, loops were defined as segments of the SA extending away from the pancreas, with a distance of 1 cm or more between the inner curve of the loop and the surface of the pancreas. SA loops were present in 80% (five out of six) of the studied specimens. The occurrence of SA loops was observed in a greater number of cases than reported in two other studies. In the study by Michels (1942), the SA was found to be straight in 15%, slightly curved in 45% and looped or coiled in 40% of the studied patients. Coiling of the SA was observed in an even lower number of subjects in the study by Sahni et al. (2003) where SA tortuosity was observed in only 10%.

Age related lengthening and widening of the SA is suggested as a cause for the development of loops in older subjects (Michels, 1942; Sahni et al., 2003). The age of the specimens of the present study was higher than in the other studies (71–100 years versus 18–80 years, respectively) and might have contributed to the finding of splenic artery loops in a greater proportion of patients. Another possible explanation for the variance between the present—and other studies is the lack of a precise definition for tortuosity, coiling or looping, resulting in variable interpretation of these parameters. Given this variability in the ages of subjects studied and the definitions used to define arterial loops, a larger sample of subjects is required to more fully address the contribution of age‐related differences in the prevalence of splenic artery loops. Given the wide spread use of cross‐sectional radiographical imaging in medical practice this would seem a means to further assess this question in vivo.

A number of potential etiologies for SA tortuosity have been suggested. These reasons were reviewed by Michels (1942) and included (a) movement and volumetric changes in the spleen require the artery to stretch, (b) tortuosity provides a damping system protecting splenic architecture, (c) tortuosity occurs during growth of the SA while it is tethered to the pancreas by its pancreatic branches, (d) tortuosity represents a developmental peculiarity which is genetically determined, and (e) tortuosity is a disease condition associated with atheroma. Little progress on our understanding on tortuosity occurrence has been made since then and so far only the last one has been ruled out as no relationship with the presence of atheroma was observed (Sylvester, Stewart, & Ellis, 1999). The results of our study argue against the hypothesis that tortuosity results from the elongation of the splenic artery between points where the SA is fixed in place by the tethering effects of PAs, with no PAs being expected to arise from with the SA loops. In our study, however, PAs were observed to originate within three out of eight SA loops and prior to SA loop intervention their presence should be carefully evaluated in order to avoid PA damage. This also applies for SGAs and the LGEA, which were observed to originate from five out of eight and two out of eight SA loops, respectively.

The SA can be divided in three segments; the prepancreatic—pancreatic and postpancreatic or perihilar segment, running between the origin of the SA and the neck of the pancreas, along the upper border of the neck and body of the pancreas and in the splenorenal ligament, respectively (Sahni et al., 2003). All SA loops observed in the present study were located at the pancreatic segment of the SA, which is consistent with earlier observations (Michels, 1942; Pandey, Bhattacharya, Mishra, & Shukla, 2004).

### 4.2 Surgical accessibility of the loops

All loops were covered with adipose and loose connective tissue, and a firm irregular network of white fibrous strands, representing nerves as confirmed by histological examination. During dissections one could easily discriminate between the SA loops and the surrounding adipose, connective and nerve tissue based on their appearance and difference in mechanical and tactile properties. Adipose tissue is yellowish and homogenous. Connective tissue is whitish, and composed of randomly organized loose fibers. Nerve plexus tissue is whitish and composed of moderate nerves, arranged in an irregular perivascular network wherein these nerves took an overall longitudinal course parallel with the SA. In only two loops were nerves observed to take a somewhat different route away from the periadventitial plexus (Figure 2a,b). These were incidental findings and were not associated with a loss of perivascular plexus, thus since they do not interfere with surgical procedures or neuroelectrode implantation on the SA loop limbs, no further attention was payed to these structures.

Nerves of the splenic plexus, which are covered by a firm perineurium, remained structurally intact and maintained their perivascular location, during arterial manipulations as part of dissection activities. In addition, these nerves are part of an irregular perivascular network which is firmly attached to the vessel wall at various locations. It was noted that when the nerves are pulled, tension is transferred to the plexus, and subsequently to the vessel wall. This results in shifting the position of the entire neurovascular bundle. This observation may be of value when the dissection is performed with laparoscopic instruments, where tactile input from the dissection instruments is reduced as compared to dissection using open techniques. While this observation has been replicated in the cadaver lab using laparoscopic dissection on fresh frozen cadavers [personal communication Irwin E.], the more formal characterization of the effects of reduced tactile input during the dissection will require further investigation. In addition, it should be noted that tissue characteristics of embalmed cadavers differ from living individuals and the ease at which the SA and its nerve plexus are dissected from its surrounding structures might differ as well. Fresh frozen cadavers represent more lifelike tissue characteristics and were used to compare these parameters. No noteworthy differences were observed [personal communication Irwin E.].

SA loops provide a space between loop limbs and the pancreas. This allows introduction of surgical instruments for the purpose of exposing the site for intervention, and, for application and stabilization of the neural interface. While these data inform the investigators involved in development of surgical techniques, further validation is required. Additional correlative radiographic and anatomical studies are in progress to meet this need. These studies will use radiographic imaging and anatomic dissections, with histological evaluation, to replicate and validate these gross anatomic findings.

### 4.3 Amount, type, and organization of nerve tissue

During dissection, perivascular white strands remained in close proximity to the wall of the loops and appeared adherent to the SA throughout the curving of the loops. These strands represent nerves of the splenic plexus as was shown by microscopic investigation of PGP9.5 stained tissue slides of SA loops. Furthermore, microscopic assessment showed these nerves to be composed of predominantly TH‐IR nerve fibers, suggesting a sympathetic nature, thereby supporting previous anatomical studies (Klein, Wilson, Dzielak, Yang, & Viveros, 1982; Nance & Burns, 1989). Evaluation of CGRP and ChAT stained tissue slides showed minor and doubtful IR for these antibodies, respectively. CGRP‐IR was observed as small dots in nerves in all specimens and its appearance morphologically consistent with nerve fibers, suggesting sensory nerve fiber presence. However, most of the staining was observed inside small blood vessels which, based on their spherical shape and their known CGRP production and secretion most likely presented lymphocytes (Wang et al., 2002).

The contribution of the of CGRP‐IR tissue in splenic plexus nerves was determined for SA loops of three different cadavers and varied from 0.64 to 1.92% of the total amount of nerve tissue. These low numbers were considered negligible, especially since the main source was non‐neural tissue. Minor ChAT‐IR areas were observed in nerve tissue of the splenic plexus. However, their morphological appearance did not clearly match nerve fibers, but instead more closely resembled cellular structures. Furthermore, ChAT‐IR was observed in blood vessel walls and in connective tissue compartments. ChAT appears to be an enzyme that is not only produced in cholinergic nerve tissue, but also in smooth muscle, endothelial and epithelial cells, and T lymphocytes (Ratcliffe et al., 1998). Additionally, this enzyme appears to be present in extracellular fluids such as plasma and CSF (Vijayaraghavan et al., 2013). Altogether, this may explain the seemingly nonspecific ChAT‐IR.

Although the spleen itself is considered to lack parasympathetic and sensory innervation (Bellinger, Lorton, Hamill, Felten, & Felten, 1993; Nance & Burns, 1989), the stomach and pancreas receive sensory and parasympathetic fibers (Kuntz, 1953; Lundberg et al., 2017) which might pass through the splenic plexus, explaining their presence in SA loop samples. To determine whether the observed sensory fibers innervate the spleen, stomach, greater omentum or pancreas, the perivascular plexus surrounding their corresponding supplying arteries is an interesting subject for future studies. Although the splenic plexus continues around SA branches towards the stomach and the greater omentum via SGAs and the LGEA, and towards the pancreas via PAs, animal studies have been performed in rodents and so far, no obvious off‐target effects have been observed (Guyot et al., 2019). Future studies in large animals and humans may provide additional information on effects on other nerves.

### 4.4 Neuroelectrode interface development

The nerves of the splenic plexus, as found in the present study, are predominantly composed of sympathetic nerve fibers. These fibers are reported to originate from the celiac ganglion and are considered to represent postganglionic unmyelinated fibers (Mitchel, 1956). The homogenous composition of nerve fibers in the nerves of the splenic plexus is favorable since less heterogeneity in electrophysiological characteristics of the various nerve fiber types is anticipated. The other parameters, such as the number of nerves, their amount of sympathetic nerve tissue, and their distance to the outer connective tissue lining of the neurovascular bundle, provide information for neuroelectrode interface development and stimulation settings. No studies which address these parameters could be found and no correlation between anatomy and function have been established. Experimental stimulation studies might provide comparative data which helps to estimate this correlation and to select therapeutic parameters that are appropriate for use in future studies in humans. Macroscopically, the splenic plexus showed the appearance of an irregular network, wrapping the SA, without clear preferential topographical orientation with respect to the dorsal, ventral, cranial or caudal sides of the vessel wall. Topographical orientation of the SA samples was not retained during tissue sampling and therefore these subjective observations could not be confirmed. Therefore, a circumferential neuroelectrode interface would address this until further studies show a preferential distribution. The median diameter of the neurovascular bundle of the SA loop limbs was determined and electrodes should be optimized to fit this dimension.

### 4.5 Limitations of the study

This study was limited to six specimens of older age and no discrimination was made between sexes. This limited data did not allow for thorough statistical analysis and therefore this study should be considered as exploratory study to characterize the feasibility of SA loops as stimulation sites for electrical SPS in humans.

## 5 CONCLUSION

SA loops and their surrounding splenic plexus could represent suitable electrical stimulation sites in the context of anti‐inflammatory therapy in humans. One or multiple SA loops are present in the majority of the studied subjects, their neurovascular bundle can be easily freed from surrounding connective and adipose tissues, and, dimensions with respect to loop height and width, provide sufficient space for introduction of surgical instruments and electrode placement. Furthermore, their surrounding splenic plexus contains a substantial amount of predominantly sympathetic nerve tissue, the target tissue for anti‐inflammatory interventions. These data provide support for further development of SPS in humans.
